# Characteristics of the school adaptation of college freshmen during the COVID-19 epidemic

**DOI:** 10.3389/fpsyg.2022.915403

**Published:** 2022-11-03

**Authors:** Hua Niu, Shuo Ren, Shuna Li

**Affiliations:** ^1^College of Marxism, Shandong University of Science and Technology, Qingdao, China; ^2^Taishan Vocational College of Nursing, Tai’an, China

**Keywords:** college freshmen, characteristics, school adaptation, COVID-19 epidemic, detection rate

## Abstract

Few studies have actually explored the impact of the COVID-19 pandemic on mental health in college students, although many studies have suggested that the COVID-19 pandemic poses a great threat to people’s mental health in many cohorts. Furthermore, college students may be a particularly vulnerable cohort that needs more attention and access to psychological services due to the psychological changes involved in the transition to college and the characteristics of college students’ study habits and lifestyle. Therefore, investigating the basic characteristics of the impact of the COVID-19 pandemic on college freshmen is of great practical importance and has theoretical implications for the identification and provisioning of services to vulnerable cohorts. A total of 5,818 college freshmen completed the College Student Adaptability Inventory. The results suggest that the mean detection rate of the seven dimensions of undergraduate maladjustment to university is 27.13%. Specifically, livelihood self-management adaptability has the highest detection rate (48.93%), while environmental general evaluation has the lowest detection rate (9.81%). Moreover, the school adaptation of college freshmen is impacted by gender, number of siblings, and family socioeconomic status (SES). Specifically, students who are female, an only child, and have a lower SES have lower levels of school adaptation. However, the school adaptation of college freshmen is not influenced by minority status or left-behind status. The findings of the present study suggest that the maladaptation of college freshmen has been a common phenomenon in China during the COVID-19 epidemic. Prevention programs may be most helpful if they pay more attention to effective intervention efforts for students who are female, an only child, and have a lower SES.

## Introduction

Due to the characteristics of rapid, extensive transmission and high infectivity, the COVID-19 pandemic has spread across the globe and poses a great threat to people’s mental and physical health. As with other major infectious disease epidemics, such as SARS, swine flu (H1N1), and avian influenza (H7N9), unexpected public crisis events can easily cause the public to experience psychological reactions, such as tension, anxiety, and even panic, which may lead to psychological disorders such as stress disorder and depression (Rajkumar, 2020; Li and Lyu, 2021). Thus, investigating the basic characteristics of the impact of the COVID-19 pandemic on public mental health and identifying and providing services for potentially vulnerable cohorts is of great practical importance and carries robust theoretical implications.

To date, many studies have investigated the impact of the COVID-19 pandemic on mental health in many cohorts, such as those of the general population (Wang et al., 2020), the affected population (Zhang et al., 2021), healthcare workers (Ricci-Cabello et al., 2020), and survivors (Zürcher et al., 2020). However, few studies have actually explored the impact of the COVID-19 pandemic on the mental health of the large and growing cohort of college students. More importantly, the cohort of college students may be a particularly vulnerable cohort that needs more attention and the provisioning of psychological services. Specifically, on the one hand, in regard to the psychological changes involved in the transition to college, the stage-environment fit theory points out that this transition is considered a major life transition (Eccles et al., 1993; Gutman and Eccles, 2007). Such a transition accompanies major developmental changes in several life spheres, including those of school, social life, and family life (Larose et al., 2019). Students must adjust to a new school environment, build new interpersonal relationships, leave the nest, and assume personal responsibility for the first time (Larose et al., 2019). The co-occurrence of these challenges may easily cause social, emotional, and learning maladjustment, resulting in difficulties in school adaptation (Leary and DeRosier, 2012; Rogers et al., 2018). Generally, school adaptation refers to the ability of students to happily participate in various activities in the school environment and to make academic progress (Ladd et al., 1997) on multiple dimensions: learning adaptability, interpersonal adaptability, social role adaptability, career choice adaptability, and other dimensions (Baker and Siryk, 1984; Lu, 2003). Numerous studies have shown that the first year is a difficult time for college freshmen (Bowman, 2010; Nightingale et al., 2013; Rogers et al., 2018). For example, Nightingale et al. (2013) found that 28.8% of college freshmen demonstrated low levels of adaptation at the beginning of enrollment. Another study found that 31.7% of freshmen show adaptive difficulties in learning, emotion, and life (Lu, 2003). Some research even found that the detection rate of maladjustment among freshmen was as high as 71.2% (Li et al., 2016). Therefore, both theoretical and empirical studies have suggested that the problem of school adaptation is widespread; that is, the cohort of college students during transition may be vulnerable to school adaptation problems.

On the other hand, regarding the characteristics of college students’ study habits and lifestyle, college students are devoted to in-person learning and high-density clustering regarding on-campus housing and recreation (Walke et al., 2020). Adhering to physical distancing is also particularly challenging for college students, for whom interaction with peers and social networks is important (Andrews et al., 2020). Such characteristics may make college students more likely to experience psychological reactions such as tension, anxiety, and even panic in the face of the rapid, extensive transmission and high infectivity of the COVID-19 pandemic. Combined with the above analyses, college freshmen are facing the dual challenges of life transition and the pressure of the COVID-19 pandemic. Based on numerous theories and models, such as the bioecological model (Bronfenbrenner’s, 1979), the allostasis model of chronic stress (McEwen’s, 1998), and developmental evolutionary theory (Ellis et al., 2009), individuals experiencing multiple risk factors are much more likely to experience psychological problems (Rutter, 1981; Evans et al., 2013). Therefore, it may be more difficult than usual to adapt to college life for college freshmen when they are exposed to the COVID-19 pandemic. Therefore, conducting a systematic investigation of the current situation and the basic characteristics of college freshmen’s school adaptation is helpful to enrich scientific research on college adaptation, improve college adaptation during the epidemic, and provide an important empirical basis for the formulation of relevant policies.

More importantly, to identify and provide services for college freshmen who are facing the dual challenges of life transition and the COVID-19 pandemic, it is necessary to investigate the potential individual differences in college student adaptation, as college student adaptation is closely related to a series of individual factors (such as gender) and social and cultural dynamics [such as family socioeconomic status (SES)]. To be specific, previous studies in developmental psychology have suggested that gender may be the most important individual factor. Theoretically, girls tend to be more concerned with pleasing others, which can enhance their efforts in school but also leave them vulnerable to increased worry about whether they are disappointing others (Pomerantz et al., 2001). Girls are more sensitive to feedback than boys and tend to view feedback as diagnostic of their ability, whereas boys tend to adopt a confident approach and deny the informational value of feedback (Roberts, 1991). As a result, male college students may have better adaptability than female college students. Indeed, previous studies have confirmed that male college students do significantly better than female college students in several dimensions of school adaptation, such as emotional adaptation (Rogers et al., 2018; Huang, 2019). Therefore, the present study hypothesizes that male college students may do significantly better than female college students in all dimensions of school adaptation during the COVID-19 pandemic.

Regarding other influencing factors, several theoretical models have been developed and have postulated that individual psychological, economic, and social resources are significantly related to the adjustment of incoming college students. For example, the individual difference resource model suggests that individuals and their families that have certain personal and social resources can effectively reduce the negative effects to their psychosocial adjustment of major life stressors (Harper et al., 2019). In this regard, college freshmen with fewer resources, such as college freshmen with siblings, minority college freshmen, and college freshmen with low SES, may have more problems with college adaptation than college freshmen with greater resources. Several studies have supported this view. For example, from the perspective of adaptation of humans to their social environment, ethnic minority college students suffer more from prejudice and other difficulties, resulting in lower school adaptation levels than non-minority college students (Credé and Niehorster, 2012). Per the family investment model and family stress model (Conger and Donnellan, 2007), because of the fewer economic, cultural, and social capital of a low-SES family, those parents invest less in their children’s development than high-SES parents, which leads to low-SES students experiencing worse academic development (Wang et al., 2017), cognitive function (Sarsour et al., 2011), and social relations (Bukowski et al., 2020). Despite its theoretical plausibility, few studies have actually conducted a detailed and systematic study of the relevant factors that affect the school adaptation of college freshmen during the COVID-19 pandemic. It is still important to factor in the impact of ethnicity, SES, and other individual and family factors. Based on the above analyses, the present study hypothesizes that college freshmen with fewer resources, including college freshmen with siblings, minority college freshmen, freshmen of left-behind status, or college freshmen with low SES, may be more likely to experience problems with college adaptation.

To summarize, in the present study, we aim to address limitations in the literature by examining the context and characteristics of the adaptation of college freshmen to school during the COVID-19 epidemic in a large sample. This study offers a more comprehensive description of the characteristics of the adaptation of college freshmen to school during the COVID-19 epidemic in mainland China, enriches previous studies with Chinese samples, promotes cross-cultural comparisons, and provides a theoretical reference for identifying and providing services for college freshmen.

## Materials and methods

### Participants

The original sample for this study comprises 5,818 college freshmen who were recruited from an engineering undergraduate university in the city of Qingdao, in Shandong Province, Eastern China. A further 97 participants were excluded from the sample due to missing data, which resulted in a final sample of 5,721 (98.33%) college freshmen (35.64% female participant and 64.36% male participant) with an average age of 18.21 years (*SD* = 0.72 years; range: 15–30 years). Data on participants’ socioeconomic status indicated that 24.9% of their mothers and 13.5% of their fathers had completed elementary school education or lower, 36.5 and 40.1% were middle school graduates, 24.0 and 18.2% were high school graduates, and 14.1 and 18.2% were college graduates or higher. In terms of employment, 76.7% of the mothers and 70.1% of the fathers were employed at working-class jobs (e.g., farmer, factory workers, and salesclerk), whereas 23.3% of the mothers and 29.9% of the fathers held a professional, managerial, or technical position (e.g., teachers, doctors, and civil servants). The sample was largely composed of working and middle-class families.

### Procedure

Participants were recruited from an engineering undergraduate university in the city of Qingdao, in Shandong Province, Eastern China. The data were collected with a mental health survey targeted at the college freshmen of Shandong University of Science and Technology. Informed consent was obtained from undergraduates during each data collection. The Institutional Review Board of Shandong University of Science and Technology approved the data collection procedures.

### Measures

#### School adaptation of college freshmen

The College Student Adaptability Inventory (CSAI, Lu, 2003) was used to assess the school adaptation of college freshmen. Sixty items are grouped into seven subscales: Learning adaptability measures students’ ability to cope with the educational demands of college (eight items, e.g., I find it hard to get used to the learning style at the university), interpersonal adaptability refers to students’ ability to build harmonious relationships (11 items, e.g., I have the ability to handle relationships with classmates independently), social role adaptability measures students’ ability to adjust their mind and behaviors independently to meet the requirements of the new role (nine items, e.g., I have the ability to adapt to changing group status), career choice adaptability represents students’ ability to manage and plan their future career choice, which is also referred to as career preparation (nine items, e.g., I have a clear career interest), livelihood self-management adaptability refers to students’ ability to organize their daily life in university without being under the care of their parents (six items, e.g., I have the ability to manage my money and plan my budget in university), environmental general adaptability quantifies a general sense of how a student feels physically and psychologically (seven items, e.g., I think the hardware facilities in our university are very poor), and somatic-mental symptom measures students’ adverse physical and psychological symptoms when completing the above adaptation tasks (10 items, e.g., I’m always out of spirits). In addition, the CSAI also provides six repeat items to verify the validity of each questionnaire. Participants rated each item on a five-point Likert scale, responding on a range from 1 = strongly disagree to 5 = strongly agree. A higher score represents a higher level of school adaptation for college freshmen.

The Chinese version of the CSAI has been widely used and has shown good internal reliability and validity (Lu, 2003; Zhang et al., 2020). In the present study, Cronbach’s α for the seven subscales ranged from 0.81 to 0.89.

#### Demographic characteristics

Demographic information was also collected from college freshmen. The information collected included the students’ gender (male/female) and age and their paternal and maternal education (elementary school or less/junior high school/senior high school/college or university/graduate or above) and the current occupation of the father and mother. Participants were also asked to complete the items related to their family composition, including their number of siblings, ethnic group, and left-behind status. Data indicated that approximately 8.98% of the participants were the only children in their family of origin, while 6.52% of the participants were left-behind children (where children may be left in the countryside to be raised by relatives while parents move to the city for work). In addition, 95.54% of the participants were Han nationality undergraduates, and 4.46% of the participants were from ethnic minority groups. The family SES of each participant was calculated by using the parents’ educational status and current occupations (Shi and Shen, 2007). Two groups of families were differentiated based on their SES scores, using the criterion of above or below the mean. As a result, 2,245 (39.24%) college freshmen were included in the high-SES group, and 3,476 (60.76%) were included in the low-SES group.

### Data analysis

SPSS 19.0 for Windows was used for data analyses. Prior to conducting analyses, any missing values were replaced with the variable mean. First, the maladjustment detection rate of school adaptation of college freshmen during the COVID-19 epidemic was calculated according to the national norms reported by previous studies (Lu, 2003). Second, a series of independent-samples *t*-tests and correlation analysis were employed to investigate whether the levels of school adaptation of college freshmen differed by undergraduates’ gender, sibling status, minority group, left-behind status, and SES. Finally, chi-square analyses were used to investigate whether the maladjustment detection rate of school adaptation differed by the aforementioned demographic characteristics.

## Results

### The maladjustment detection rate of school adaptation of college freshmen

The maladjustment detection rates of school adaptation of college freshmen during the COVID-19 epidemic were calculated according to the national norms for the school adaptation of Chinese undergraduates reported in previous studies (Lu, 2003). As shown in Table 1, the mean of the maladjustment detection rates of the seven dimensions of school adaptation of college freshmen was 27.13%. Specifically, livelihood self-management adaptability showed the highest detection rate (48.93%), and environmental general evaluation showed the lowest detection rate (9.81%). In other words, Chinese college freshmen demonstrated the lowest level of school adaptation in livelihood self-management adaptability and demonstrated the highest level of school adaptation in environmental general evaluation.

**TABLE 1:** The mean (M), standard deviation (SD), and detection rate of undergraduates’ maladjustment to university.

	*M* ± *SD*	Detection rate (%)
Learning adaptability	29.49 ± 6.25	27.64
Interpersonal adaptability	43.48 ± 7.10	28.18
Social role adaptability	36.44 ± 5.60	28.53
Career choice adaptability	30.10 ± 6.74	21.27
Livelihood self-management adaptability	25.31 ± 3.41	48.93
Environmental general adaptability	27.80 ± 4.60	9.81
Somatic-mental symptom	40.54 ± 6.57	25.52

### The effect of gender

A series of independent-samples *t*-tests were employed to investigate whether the levels of school adaptation of college freshmen differed by students’ gender. As shown in Table 2, compared to female freshmen, male freshmen demonstrated significantly higher levels of school adaptation in career choice adaptability (*t* = 2.47, *p* < 0.05) and environmental general adaptability (*t* = 2.38, *p* < 0.05) but not in the other five dimensions of school adaptation (*t*s < 1.17, *p* > 0.05). The chi-square analyses found no significant gender difference in the maladjustment detection rates of seven dimensions of school adaptation of college freshmen (χ^2^ < 1.87, *p* > 0.05).

**TABLE 2:** The differences between male and female undergraduates on primary study variables.

	*M* ± *SD*			Detection rate (%)		
		
	Male	Female	*t*	Male	Female	χ ^2^
Learning adaptability	29.55 ± 6.31	29.39 ± 6.12	0.97	27.54	27.81	0.05
Interpersonal adaptability	43.49 ± 7.24	43.43 ± 6.86	0.27	28.10	28.22	0.01
Social role adaptability	36.49 ± 5.66	36.35 ± 5.49	0.95	28.15	28.73	0.22
Career choice adaptability	30.26 ± 6.80	29.80 ± 6.62	2.47*	22.27	20.72	1.87
Livelihood self-management adaptability	25.35 ± 3.43	25.24 ± 3.38	1.17	49.49	48.61	0.40
Environmental general adaptability	27.90 ± 4.59	27.60 ± 4.61	2.38*	10.45	9.45	1.47
Somatic-mental symptom	40.60 ± 6.60	40.43 ± 6.51	0.90	25.70	25.42	0.05

*p < 0.05.

### The effect of the number of siblings

A series of independent-samples *t*-tests were employed to investigate whether the levels of school adaptation of college freshmen differed by number of siblings. As shown in Table 3, compared to freshmen with siblings, those without siblings demonstrated a significantly lower level of school adaptation in livelihood self-management adaptability (*t* =−2.26, *p* < 0.05), environmental general adaptability (*t* =−4.13, *p* < 0.001), and somatic-mental symptoms (*t* =−2.61, *p* < 0.05) but not in the other four dimensions of school adaptation (*t*s < 1.54, *p* > 0.05). The chi-square analyses also found that the maladjustment detection rates of livelihood self-management adaptability (χ^2^ = 7.64, *p* < 0.01) and somatic-mental symptoms (χ^2^ = 3.94, *p* < 0.05) were higher in freshmen without siblings than in freshmen with siblings but not in the other five dimensions.

**TABLE 3:** The differences between only child and children with siblings undergraduates on primary study variables.

	*M* ± *SD*			Detection rate (%)		
		
	Only child	Children with siblings	*t*	Only child	Children with siblings	χ ^2^
Learning adaptability	29.35 ± 6.29	29.59 ± 6.21	–1.40	27.53	27.70	0.02
Interpersonal adaptability	43.29 ± 7.13	43.58 ± 7.08	–1.54	29.24	27.50	2.03
Social role adaptability	36.34 ± 5.65	36.51 ± 5.57	–1.15	29.96	27.61	3.66
Career choice adaptability	30.00 ± 6.76	30.16 ± 6.72	–0.84	20.81	21.57	0.47
Livelihood self-management adaptability	25.18 ± 3.44	25.39 ± 3.39	−2.26*	51.21	47.46	7.64**
Environmental general adaptability	27.48 ± 4.71	28.00 ± 4.51	−4.13***	10.76	9.20	3.78
Somatic-mental symptom	40.26 ± 6.67	40.72 ± 6.50	−2.61**	26.95	24.61	3.94*

*p < 0.05. **p < 0.01. ***p < 0.001.

### The effect of socioeconomic status

As shown in Table 4, correlation analyses suggested that SES was significantly related to social role adaptability, career choice adaptability, livelihood self-management adaptability, and environmental general adaptability (*r*s ≥−0.03, *p*s < 0.05). The chi-square analyses also found that the maladjustment detection rates of environmental general adaptability (χ^2^ = 4.72, *p* < 0.05) were lower in low-SES freshmen than in high-SES freshmen but not in the other six dimensions.

**TABLE 4:** The relations between primary study variables and socioeconomic status (SES).

	*r*	Detection rate (%)		
		
		Low SES	High SES	χ ^2^
Learning adaptability	−0.02	27.33	28.11	0.41
Interpersonal adaptability	−0.02	27.79	28.78	0.65
Social role adaptability	−0.03*	27.73	29.76	2.74
Career choice adaptability	−0.03*	21.29	21.25	0.00
Livelihood self-management adaptability	−0.03*	48.71	49.27	0.17
Environmental general adaptability	−0.05**	9.12	10.87	4.72*
Somatic-mental symptom	−0.02	24.86	26.55	2.05

*p < 0.05. **p < 0.01.

### The effect of other factors

As shown in Tables 5, 6, a series of independent-samples *t*-tests and chi-square analyses found that both the levels and the maladjustment detection rate of school adaptation of college freshmen did not differ by students’ ethnic group and left-behind status.

**TABLE 5:** The differences between Han nationality and minority group undergraduates on primary study variables.

	*M* ± *SD*			Detection rate (%)		
		
	Han nationality	Minority group	*t*	Han nationality	Minority group	χ ^2^
Learning adaptability	29.50 ± 6.25	29.38 ± 6.11	0.30	27.63	27.84	0.01
Interpersonal adaptability	43.48 ± 7.08	43.30 ± 7.66	0.35	28.08	30.20	0.54
Social role adaptability	36.44 ± 5.60	36.44 ± 5.60	0.01	28.45	30.20	0.37
Career choice adaptability	30.08 ± 6.72	30.44 ± 7.05	−0.83	21.35	19.61	0.44
Livelihood self-management adaptability	25.31 ± 3.40	25.29 ± 3.64	0.11	49.01	47.06	0.37
Environmental general adaptability	27.80 ± 4.59	27.73 ± 4.73	0.24	9.79	10.20	0.05
Somatic-mental symptom	40.53 ± 6.56	40.63 ± 6.89	−0.23	25.50	25.88	0.02

**TABLE 6:** The differences between left-behind and non-left-behind undergraduates on primary study variables.

	*M* ± *SD*			Detection rate (%)		
		
	Left-behind	Non-left-behind	*t*	Left-behind	Non-left-behind	χ ^2^
Learning adaptability	29.71 ± 6.08	29.48 ± 6.26	0.69	24.67	27.84	1.76
Interpersonal adaptability	43.60 ± 6.96	43.46 ± 7.11	0.37	30.56	28.01	1.12
Social role adaptability	36.63 ± 5.46	36.43 ± 5.61	0.68	27.61	28.59	0.16
Career choice adaptability	30.10 ± 6.59	30.10 ± 6.75	0.01	21.72	21.24	0.05
Livelihood self-management adaptability	25.62 ± 3.23	25.29 ± 3.42	1.80	45.84	49.14	1.52
Environmental general adaptability	28.00 ± 4.71	27.78 ± 4.59	0.89	9.92	9.80	0.01
Somatic-mental symptom	41.09 ± 6.14	40.50 ± 6.60	1.69	24.13	25.62	0.41

## Discussion

### The maladjustment detection rate of school adaptation of college freshmen

The present study expanded on previous research by investigating the current context and basic characteristics of school adaptation of college freshmen during the COVID-19 epidemic. To the best of our knowledge, the present study is the first large-scale survey to explore these issues, and the results are highly representative. The results demonstrated that livelihood self-management adaptability was the most difficult aspect for college freshmen during the COVID-19 epidemic, with a maladjustment detection rate of school adaptation as high as 48.93%. Nearly half of college freshmen had difficulties with self-care, which made it difficult to adapt to college life. The first possible reason may be attributed to the current situation of family education in China. Chinese parents place a high value on their children’s study time and may be more willing to provide work for their children in terms of clothing, food, housing, and transportation. As a result, Chinese college freshmen lack life skills and have difficulty quickly adapting to self-disciplined college life. Second, the social and cultural changes brought by economic and social development in China have increasingly strengthened the individualism value among Chinese college students, whereas the collectivism values have gradually weakened (Kashima et al., 2011; Hamamura, 2012; Liu et al., 2020). College freshmen may find it difficult to accommodate each other when they live collectively for the first time, which results in lower levels of livelihood self-management adaptability. Finally, due to the high infectivity of COVID-19, college freshmen have varying degrees of anxiety and panic about the high intensity of collective life, which may also result in lower levels of livelihood self-management adaptability.

In addition, the average maladjustment detection rate of seven aspects of school adaptation was 27.13%. This result was lower than the maladjustment detection rate of school adaptation among Chinese college students in previous studies, such as that of 31.7% in Lu (2003), 48% in Cai (2013), 57.2% in Guo et al. (2014), and 71.2% in Li et al. (2016). Although the previous studies cannot be directly compared with the present study, the research tools and test samples were different. Considering that the college freshmen in the present study were facing the dual challenges of life transition and the pressure of the COVID-19 pandemic, it can be said that the school adaptation level of college students in China is gradually getting better to a certain extent. However, there were still high maladjustment detection rates of school adaptation of college freshmen in all aspects during the COVID-19 epidemic. Considering that, like China, numerous countries are facing the challenges of major infectious disease epidemics, such as the COVID-19 epidemic and the monkeypox virus, college freshmen around the world are facing the dual challenges of life transition and the pandemic. Thus, the present study offers a more comprehensive description of the characteristics of school adaptation during a major infectious disease, enriches previous studies with Chinese samples, promotes cross-cultural comparisons, and provides a theoretical reference for identifying and providing services for college freshmen. Future research should conduct more in-depth and systematic research on the characteristics of school adaptation development, its relevant influencing factors, and potential intervention strategies, which are of great importance in improving the cultivation of talent at universities.

### The effect of gender

Previous studies revealed that male college freshmen do significantly better than female college freshmen in adapting to school in many aspects (Rogers et al., 2018; Huang, 2019). Partially consistent with previous studies, the present study found that male freshmen do significantly better than female freshmen in career choice adaptability and environmental general adaptability, although there was no significant gender difference in the other five aspects. One possible reason is differing parental expectations concerning the roles of boys and girls (Maccoby and Jacklin, 1974; Mathur and Berndt, 2006; Hibbard and Buhrmester, 2010). Specifically, parents generally teach boys to be more independent and stronger, which encourages men to constantly adopt these characteristics. Compared with girls, boys have to adjust their psychological state to adapt to the new environment as soon as possible to meet this internal role expectation (Huang, 2019). Another possible reason may be the traditional Chinese belief that “a man is afraid of going in the wrong direction.” In this regard, compared to girls, Chinese parents may pay more attention to boys’ choices of college majors and may be more likely to make boys think about their future career options. Thus, compared with female freshmen, male freshmen may pay more attention to their career planning and may have better psychological preparation when applying for college, which leads them to have better performance in career choice adaptability and environmental general adaptability. The results suggested that parents should abandon gender stereotypes and provide more care and help for girls’ career development. Likewise, schools should also provide more guidance and advice regarding this issue.

### The effect of the number of siblings

According to the resource dilution model, the resources a parent can provide for a particular child decrease as the number of children increases (Blake, 1981). Parents tend to invest more time, money, and energy in their only child. As the sole undertaker in the family, the only child is more likely to be affected by the parent-child relationship (Chen and Liu, 2014; Xing et al., 2019). This may result in the only child being more dependent on the parent-child relationship and thus less independent. Children with siblings, on the contrary, come from more complex families, where interactions with siblings may lead them to learn social skills earlier and become more social. Previous studies have found that children with siblings do significantly better at independent living and self-management than children with no siblings (Yang et al., 2003). Similarly, the results of this study also found that children with siblings adapted significantly better than children with no siblings in the areas of livelihood self-management adaptability, environmental general adaptability, and somatic-mental symptoms. The current results suggested that since the fifth Plenary Session of the 18th CPC Central Committee decided to fully implement the “two-child” policy, this policy was not only of great importance in alleviating labor shortages and reducing the burden of the aging population but also had a positive impact on individual social development. However, there are still few studies on this issue in China, and the deep mechanism behind the differences between multiple-child households and single-child households still needs further discussion.

### The effect of socioeconomic status

The family investment model and family stress model (Conger and Donnellan, 2007) both point out that the economic, cultural, and social capital of a family determines the levels of parents’ investment in children’s development and thus directly affects children’s development. Empirical studies also found that low SES has a demonstrated negative effect on individual academic development (Wang et al., 2017), cognitive function (Sarsour et al., 2011), and social relations (Bukowski et al., 2020). However, in contrast to a previous study and our initial expectations, the results of the present study found a significant negative correlation between SES and college adaptation. That is, college freshmen with low SES showed better adaptation at school in social role adaptability, career choice adaptability, livelihood self-management adaptability, and environmental general adaptability. College freshmen with low SES also had a lower maladjustment detection rate than college freshmen with high SES in environmental general adaptability. This may be because the parents of freshmen with low SES need to invest more energy in managing the household and thus invest less direct management of their children, which leads to a better self-care ability for these freshmen (Yang et al., 2003). As a result, college freshmen with low SES had better adaptability in four aspects related to life, while there was no difference in learning or the other aspects.

However, it should be noted that this finding does not mean that low SES has a positive impact on the development of college freshmen. On the one hand, the positive effects found in this study were only seen in several life aspects when entering university, whereas there was no significant advantage in learning or other aspects. With increased familiarity with college life, this slight advantage may disappear. On the other hand, the sample SES of this study was relatively concentrated, and there were few college freshmen with either extremely low SES or extremely high SES. Therefore, the negative impact of extremely low-SES families on individual development may be masked by sampling problems. In future, it is necessary to expand the sample representation, further refine school adaptation indicators, and investigate the influence of SES on college adaptation of college freshmen in an in-depth and systematic way.

### The effect of other factors

This study also investigates the impact of ethnic group and left-behind status on school adaptation. This study found that neither ethnic group nor left-behind status had a significant effect on the school adaptation of college freshmen. On the one hand, this result shows that China has attached great importance to ethnic cultural integration and the development of left-behind children in recent years, which has reduced the negative impact of ethnic minorities and left-behind status by providing a high-quality platform and environment for these college students. On the other hand, the influence of ethnic group or left-behind status on individual development may exist in other aspects that were not examined in this study, so it is yet of great significance to conduct a more extensive investigation and further research.

### Limitations and implications for interventions and future research

Several limitations of this study should be noted. First, given that this study was a cross-sectional study, it is impossible to reveal the characteristics of the development trajectory of the school adaptation of college freshmen over a long period of time. Future studies will require prospective longitudinal data across a considerable length of time. Second, the participants in the present study were recruited from a representative technological university, which led to a large percentage of the participants being male. Thus, caution should be taken in the conclusions drawn about the characteristics of the school adaptation of college freshmen during the COVID-19 epidemic. Finally, the participants in this study were primarily from the Han nationality, non-left-behind experience family. Future research is necessary to investigate the characteristics of the school adaptation of college freshmen across a more representative sample.

Despite these limitations, some important practical implications and valuable information can be derived from the present study. On the one hand, this study offers a more comprehensive description of the characteristics of the adaptation of college freshmen to school during the COVID-19 epidemic in mainland China, enriches previous studies with Chinese samples, promotes cross-cultural comparisons, and provides a theoretical reference for identifying and providing services for college freshmen. The results suggested that the mean maladjustment detection rate of the seven dimensions of school adaptation of college freshmen is 27.13%. Specifically, livelihood self-management adaptability shows the highest maladjustment detection rate, and environmental general evaluation shows the lowest maladjustment detection rate. These findings highlight that in education practice, it is necessary to provide more psychosocial services in the school adaptation of college freshmen, especially regarding livelihood self-management adaptability. On the other hand, the present study systematically reveals the characteristics of the school adaptation of college freshmen and provides a theoretical reference for identifying and providing services for college freshmen. Specifically, female, only child, and lower SES freshmen showed lower levels of school adaptation. Thus, more attention should be given to effective intervention efforts for vulnerable freshmen groups, including freshmen who are female, only children, and have a lower SES.

## Data availability statement

The data analyzed in this study is subject to the following licenses/restrictions: The research group keeps the data confidential. Requests to access these datasets should be directed to HN, niuhua_890209@126.com.

## Ethics statement

The studies involving human participants were reviewed and approved by Institutional Review Board of Shandong University of Science and Technology. The patients/participants provided their written informed consent to participate in this study.

## Author contributions

HN was responsible for writing the manuscript. SR was in charge of data processing. SL was responsible for the research design. All authors contributed to the article and approved the submitted version.
